# Properties of Polyhexamethylene Guanidine (PHMG) Associated with Fatal Lung Injury in Korea

**DOI:** 10.3390/molecules25143301

**Published:** 2020-07-21

**Authors:** Dong-Uk Park, Jihoon Park, Kee Won Yang, Ju-Hyun Park, Jung-Hwan Kwon, Han Bin Oh

**Affiliations:** 1 Department of Environmental Health, Korea National Open University, Seoul 03087, Korea; 2 Environmental Safety Group, Korea Institute of Science and Technology Europe Forschungsgesellschaft mbH, 66123 Saarbrücken, Germany; jhabso@nate.com; 3 Institute of Health and Environment, Graduate School of Public Health, Seoul National University, Seoul 08826, Korea; 4 Department of Chemistry, Sogang University, Seoul 04107, Korea; scr1109@gmail.com; 5 Department of Statistics, Dongguk University, Seoul 04620, Korea; juhyunp@gmail.com; 6 Division of Environmental Science and Ecological Engineering, Korea University, Seoul 02841, Korea; junghwankwon@korea.ac.kr

**Keywords:** humidifier disinfectant-associated lung injury, PHMG, oligomer, molecular mass, polymerization

## Abstract

The use of humidifier disinfectant (HD) has been determined to be associated with lung injuries (HDLI) in Korea. Although HD brands containing polyhexamethylene guanidine (PHMG) oligomers have been found to cause more HDLI compared to brands containing other disinfectants, the physicochemical properties of PHMG have been poorly defined. We aimed to quantify the PHMG dissolved in HD brands, characterize the number-average (*M_n_*) and weight-average (*M_w_*) molecular masses, and identify the polymerization degree of PHMG. Analysis of the PHMG oligomers was performed using a matrix-assisted laser desorption/ionization time-of-flight mass spectrometer (MALDI-TOF MS) operated in positive-ion reflectron mode. Eight brands of HD containing PHMG were identified. The PHMG concentrations in these brands ranged from 160 to 37,200 ppm (mean = 3100.9 ppm). Concentration was a significant variable among and within HD brands. The degree of PHMG oligomerization fell within the range of two to four. The averages of *M_n_* and *M_w_* were 517.2 g/mol (range: 422–613 g/mol) and 537.3 g/mol (range: 441.0–678.0 g/mol), respectively. Based on the average molecular weight and the degree of polymerization, the PHMG examined here could be regarded as oligomers, which may be associated with the highest proportion of HDLI being caused by PHMG.

## 1. Introduction

Several types of chemicals were widely used as humidifier disinfectants (HD) in South Korea from 1994 until the end of 2011. The use of HD brands containing polyhexamethylene guanidine (PHMG), oligo (2-(2-ethoxy) ethoxyethyl guanidinium (PGH), and a mixture of chloromethylisothiazolinone (CMIT) and methylisothiazolinone (MIT) has been confirmed to be associated with lung injury, including interstitial pneumonitis and widespread lung fibrosis. Collectively, this has been named humidifier-disinfectant-associated lung injury (HDLI) [1,2,3]. Several studies have demonstrated that chemicals added to a humidifier’s water tank as a disinfectant in order to suppress microbial growth have caused fatal HDLI in children, pregnant women, and even adults [1,3]. From May 2013 to date, the government has operated the Humidifier-Associated Lung Injury Investigation and Decision Committee (HLIIDC) to evaluate whether registered patients are clinically associated with HD use [4]. Among 221 HDLI patients clinically examined through two rounds of investigations conducted from July 2013 until April 2015, a total of 123 (55.7%) used HD products containing PHMG [4]. Even though HD products containing PHMG were found to cause the highest number of HDLI compared to HD brands using other disinfectants, the physicochemical properties of PHMG as used in the HDs have been poorly defined.

**Figure 1:**
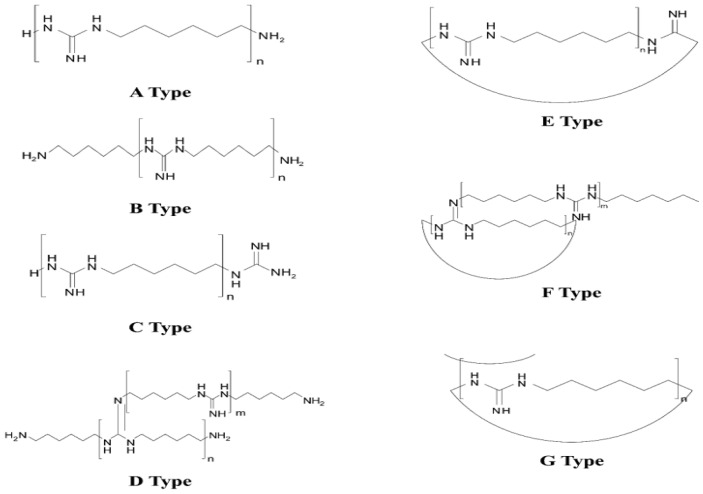
Structures of polyhexamethylene guanidine (PHMG) isomers. Depending on the polymer (oligomer) end groups and the connectivity between the two end groups, PHMG can take on different isomeric forms [11,12].

PHMG is a family of polymers containing guanidine subunits (Figure 1) that has been proven to be highly biocidal for a range of microorganisms while showing low toxicity to humans [5,6,7]. It has been regarded as a safe alternative to common disinfectants such as formaldehyde, ethylene oxide, chlorine, hypochlorite solutions, iodine, alcohols, phenols, or other compounds, and has undergone widespread application in all areas of human life as a nontoxic disinfectant or additive [8,9,10]. Biocidal cationic polymers, including PHMG, have attracted considerable attention for their high antibacterial activity and low human toxicity.

The objectives of this study are to quantify the concentrations of PHMG dissolved in HD brands and characterize the molecular weight and the degree of polymerization of PHMG. The results can be used to evaluate the effect of PHMG on the development of diseases, including lung injury.

## 2. Results

**Figure 2:**
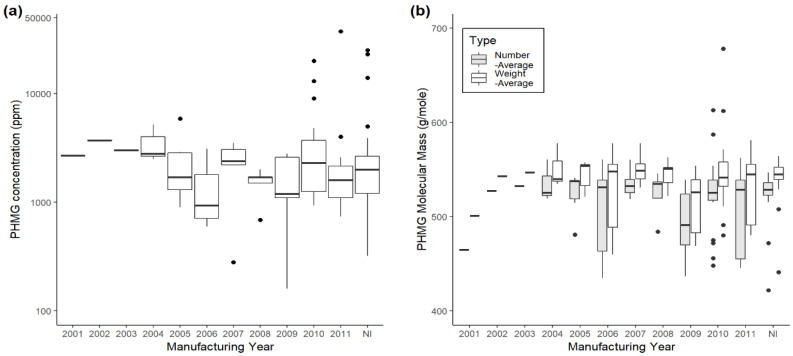
Boxplots of (**a**) PHMG concentration (ppm) on the log scale and (**b**) molecular mass (g/mol) contained in the humidifier disinfectant (HD) products by year manufactured. Note: NI indicates “no information”.

To date, a total of eight HD brands have been identified as containing PHMG. Oxy Saksak was manufactured as an HD from 2000 through the end of 2011, accounting for the majority of the market volume in Korea (Table 1). The sales volumes of other HD brands have not been identified, but would likely be minimal compared to Oxy Saksak. The concentrations of PHMG in HD brands ranged from 160 to 37,200 ppm (mean = 3100.9 ppm). The concentration variation was significant both among and within HD brands, and also for products manufactured in different years within the same HD brand. However, specific trends within the PHMG concentration, year manufactured, and HD brand were not observed (Table 2 and Figure 2). Our results indicate that the degree of PHMG oligomerization was in the range of two to four. The averages of *M_n_* and *M_w_* were, respectively, found to be 517.2 (g/mol), with a range of 422–613 (g/mol); and 537.3 (g/mol), with a range of 441.0–678.0 (g/mol). The *M_w_*’s were slightly higher than *M_n_*’s (Table 3 and Figure 2). Based on the average molecular weight and degree of polymerization, the PHMGs examined here could be regarded as oligomers (Table 4).

## 3. Discussion

We described the physicochemical properties of PHMG based on its concentration, molecular weight, and degree of polymerization (Table 1, Table 2 and Table 3). The PHMG analyzed in this study contained oligomer compounds of relatively low molecular weight (*M_w_*), containing up to four monomer units. In addition, a monomer of hexamethylenediamine (HMDA), which did not react during the PHMG polymerization reactions, was found to remain within the range of a significant quantity. According to the Korean Centers for Disease Control and Prevention (KCDC) report that the average size of HD aerosol dispersed into the air ranged from 30 nm to 80 nm in a 1500 L chamber using an ultrasonic humidifier and monitored by a scanning mobility particle sizer (SMPS) [14], most of the inhaled PHMG dose can likely easily penetrate into the alveoli region of the respiratory system, the target organ injured by HD [15]. Because of the complex properties of PHMG, other physicochemical properties, such as the molecular mass, degree of polymerization, and concentration of HMDA, may be related to the level of toxicity. We will discuss how these properties of PHMG might be associated with health problems, including HDLI.

Firstly, the PHMG concentrations dissolved in HD brands were found to be far higher than the minimal inhibition concentration (MIC) of PHMG (range: 0.78–25 ppm) determined by testing for good antimicrobial activity against several different microorganisms, including bacteria and fungi [16,17,18]. PHMG has been reported to kill methicillin-resistant *Staphylococcus aureus* and *Escherichia coli* at concentrations as low as 0.04 and 0.005% (*w/v*), respectively, within 1.5 min [19]. Choi et al. reported that PHMG exerts antifungal activity against various fungal strains in the range of 1.25–2.5 μg/mL (ppm) [20]. These results suggest that PHMG induces membrane depolarization, resulting in the loss of membrane functions and leading to cell death. Zhou et al. reported that the inactivation effect of PHMG had a dose, time, and inoculum concentration [21]. They found the MIC value of PHMG to be 4 ppm, the lowest concentration of this antimicrobial that totally inhibited macroscopically visible growth of the inoculum. This result showed that a low concentration of PHMG mainly damaged the outer membrane structure and that no significant damage to the intracellular structure was observed. In contrast, after exposure to a high concentration of PHMG, although the general morphological structure of the cells was retained, the integrity of the cell wall layer structure was destroyed (mostly through collapse) and obvious gaps could be seen in some cells [21]. If 20 mL of PHMG at a concentration of 3073 ppm is injected into 2 L of humidifier water, the estimated level of PHMG in the humidifier would be 30.7 ppm. Most of the levels of PHMG estimated based on average PHMG use and frequency per day could be higher than the MIC reported elsewhere [19,21].

Secondly, there may be a possibility that the molecular mass of PHMG is related to health problems, including HDLI. The molecular weight of PHMG used for identifying MIC has been around 1000 Daltons (Da). However, the number and weight of the average molecular masses of PHMG in the HD samples investigated in the current research were 517.2 g/mol (*M_n_*) and 537.3 g/mol (*M_w_*) (Table 3 and Figure 2), respectively, far lower than those required for an efficient antimicrobial activity assay (up to 800 Da) [22]. Interestingly, the molecular masses among various HD brands were similar, despite the differences in concentrations between and within HD brands. Wei et al. reported that an aqueous solution of PHMG (*M_w_* = 640) at a concentration as low as 1.0 ppm exhibited an antibacterial rate above 90.0% [11]. At least a significant portion of the commercially available oligomeric PHMG seems to be in the form of an oligomer with a lower degree of polymerization, which could not be classified as a “polymer” under the regulatory framework [23,24]. To our knowledge, the effect of the molecular weight of PHMG on health risks has not been examined. In general, components with a molecular mass above 1000 Da are known to be very unlikely to be absorbed by the gastrointestinal tract, and thus are not considered to present a toxicological risk. The value of 1000 Da was chosen because it takes into account the effect of the shape of the molecule, which has an important influence on the likelihood of absorption of substances in the molecular mass range of 600–1000 Da. Below 600 Da most substances are absorbed, and the rate of absorption is determined by factors other than the size and shape of the molecule [25]. Albert et al. concluded that lower *M_w_*’s (800) result in a rapid decrease of activity [22]. When increasing the chain length of the diamine, the biocidal activity drops accordingly. Regarding the parameters that have an influence on biocidal activity, it can be shown that the *M_w_* of oligoguanides has to be in the range of 800–1300 Da. The weight percentage of oligomers with a molecular weight < 500 must be less than 5% in the USA and China and 2% in Japan and Korea [24].

In 1997, PHMG was registered as an existing chemical without any evaluation of its inhalation toxicity under the Toxic Substances Control Law (TSCL) of Korea (enacted in 1991), because the usage of PHMG as a humidifier disinfectant was not clearly defined [26]. PHMG was also registered in Australia by a major supplier from Korea, with the announced use as a microbial additive in plastics, fabric softeners, paints, swimming pools, and paper, as well as for sanitation in food processing plants and cooling towers. It was registered as a polymer with a measured average molecular weight number (*M_n_*) and average molecular weight (*M_w_*) of 18,500 and 137,000 g/mol, respectively [27]. The reported *M_n_* and *M_w_* values are much greater than those characterized in this study. Although the method of molecular weight determination was not specified in the National Industrial Chemicals Notification and Assessment Scheme (NICNAS) report, it is likely that gel permeation chromatography (GPC) was used, because the supplier submitted GPC results in Korea. Because polystyrene-equivalent *M_w_* obtained by GPC includes greater uncertainties [28], the current result of a much lower average molecular weight should be given priority over those previously reported.

Thirdly, the level of PHMG oligomers and different *M_w_* values may be associated with toxicity, including antibacterial activities. Oligomers 3 and 7 have approximately the same antibacterial activity as benzalkonium chloride (BAC) [22]. Ionic interactions of PHMG with head groups of cell membranes were found to be dominant in the distribution of PHMG between solid-supported lipid membranes and water [29]. However, PHMG also accumulated in an example membrane with cationic head groups (1,2-dioleoyl-3-trimethylammonium-propane, DOTAP), implying that the positively charged guanidine group of PHMG may align with the cationic head of the DOTAP lipid, leading to membrane disruption and pore formation [29]. Although further investigation is needed, it is probable that PHMG may enter the cell and interact with intracellular components after adherence to the membrane. The prevailing model for PHMG activity holds that guanidine kills bacteria through bacterial membrane damage, and that the polymer does not interact with mammalian cell membranes. To our knowledge, there has been no study to test how the characteristics of the PHMG oligomers found in this study associate with antimicrobial activity, including the degree of cell membrane permeation. We cannot rule out the possibility that PHMG from HD aerosols was deposited in the lung airway and that the active ingredient PHMG permeated the epithelial barriers. This merits further studies on cell membrane permeability and related toxic mechanisms with the precise characterization of PHMG used in HDs. Further study is needed to evaluate whether differences between two counter ions of PHMG (phosphate and hydro chloride) are related to toxicity. No HDLI patient among the people who used only PHMG with hydrochloride has been reported to date, even though the concentrations used are far higher than those of PHMG-phosphate [30].

Finally, the level of hexamethylenediamine (HMDA) remaining unreacted as a monomer in HD brands may contribute to the development of HDLI. HMDA can cause irritation of the skin, eyes, and other mucous membranes in humans, as well as in the upper respiratory tract [31]. Park et al. estimated the airborne HMDA levels based on the dissolved concentration (n = 16, range: 8.61 to 49.7 ppm, mean: 19.4 ppm) and HD use characteristics from five HD brands containing PHMG [13]. The levels of airborne HMDA ranged from 0.48 to 16.40 μg/m^3^, which were higher than the 1.8 μg/m^3^ value for respirable particles with diameters of 10 μm and smaller [32]. Repeated exposure inhalation studies have defined the upper respiratory tract to be the first target of HMDA. The irritation seen is proportionate to the exposure concentration [31].

## 4. Materials and Methods

### 4.1. Collection of Humidifier Disinfectant (HD) Samples

All HD samples were collected from people who registered with the HLIIDC program during the environment investigation visit. Samples were stored in PE bottles, transported by ice box, and stored in a refrigerator. A total of 111 samples from HD brands that were assumed to contain PHMG were analyzed in order to characterize the physicochemical properties of the PHMG, such as the concentration, number-average (*M_n_*) and weight-average (*M_w_*) molecular masses, and the degree of polymerization. Samples with concentrations below the limit of quantitation (LOQ) for PHMG were excluded. The number of samples by HD brand were found to be severely unbalanced because of the considerable differences in sales volumes. The methods applied to evaluate the use characteristics of HDs based on personal interviews and home investigations have been described elsewhere [30,33]. Trained environmental health scientists visited registered patients’ homes and conducted personal interviews and home investigations with the patients and their family members to complete detailed questionnaires or checklists related to HD use.

### 4.2. Quantification of PHMG

Analysis of the PHMG oligomers was made using a matrix-assisted laser desorption/ionization time-of-flight mass spectrometer (MALDI-TOF MS, Autoflex series, Bruker Daltonics, Bremen, Germany) operated in the positive-ion reflectron mode. The experimental procedure is described in detail elsewhere [12,34,35]. Initially, qualitative analysis was performed to identify the presence of guanidine-containing oligomers. A sample was purified and enriched using mixed-mode solid-phase extraction (MCX SPE, 1 mL, Waters; Milford, MA, USA). The MCX SPE cartridge consisted of a strong cation exchange and reversed-phase sorbent materials. The activation of the MCX cartridge was performed by eluting 1 mL of methanol (Fisher Scientific, Waltham, MA, USA) three times, and then pre-wetted by elution with 1 mL of a 0.1% HCl/methanol solution (pH = 2–3) three times; the HCl was purchased from Daejung (36.0–38.0% *w/w*, Goryeong, Gyeongsangbuk-do Province, Korea). A sample was loaded by eluting 1 mL of the analyte solution through the solid-phase extraction (SPE) cartridge. This cartridge was washed by eluting 1 mL of 0.1% HCl/methanol solution three times. Finally, the guanidine-containing oligomers were eluted by 1 mL of a 2 M HCl/methanol (pH = 1–2) solution. The eluted analyte solution was dried by nitrogen blowing at the 1 mL Eppendorf tube. Water of high-performance liquid chromatography (HPLC) grade (Burdick and Jackson, Morristown, NJ, USA) was added into the Eppendorf tube to produce a 1 mL final solution.

Through solid-phase extraction, other matrix materials could be eliminated and the guanidine-containing oligomers were selectively enriched. The purified sample was mixed with an ionic liquid matrix (ILM) composed of α-Cyano-4-hydroxycinnamic acid (CHCA, CAS No. 28166-41-8, Sigma, St. Louis, MO, USA) and 1-methyl-imidazole (CAS No. 616-47-7, Sigma, St. Louis, MO, USA). A 1 μL aliquot of the mixed ILM sample solution was deposited onto a MALDI plate, wherein a homogeneous thin film of the mixed material was formed. The homogeneous thin film ensured a reproducible detection of the analytes under examination. The MALDI mass spectra were acquired by irradiating a 355 nm laser light operating at 500 Hz. Five different points within the single MALDI plate spot were sampled using 1000 laser shots to get a single MALDI mass spectrum. The acquired MALDI mass spectra were examined to see whether there are peaks corresponding to the *m*/*z* values of the oligomer series and their isomers. The operation parameters for the MALDI-TOF MS were as follows: ion-source 1 voltage, +19.05 kV; ion-source 2 voltage, +16.70 kV; laser power percentage, 48%; pulsed ion extraction, 140 ns; lens voltage, +8.24 kV; reflector voltage, +20.99 kV; and reflector 2 voltage, +9.73 kV.

Once the guanidine-containing oligomers were identified, quantitative analysis was made by comparing the abundance of the detected peaks with those of the corresponding internal isotope peaks. First, a calibration plot was constructed using the standard ^12^C and reference (internal standard) ^13^C-labeled counterpart guanidine oligomers (FutureChem, Seoul, Korea). Then, 1000 ppm of the internal standard of the identified oligomer was spiked into the sample under investigation. Thus, the sample and the internal standard material were subjected to the same sample purification and quantitative measurement procedure to ensure accurate quantitation.

### 4.3. Detection of the Guanidine Oligomers

**Figure 3:**
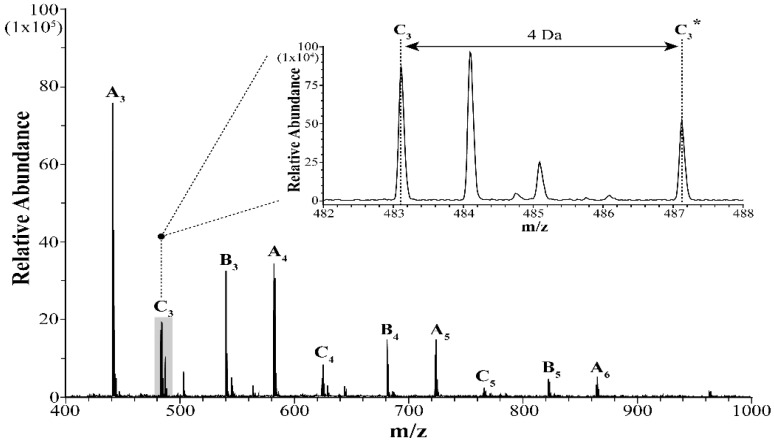
A representative matrix-assisted laser desorption/ionization time-of-flight mass spectrometer (MALDI-TOF MS) spectrum in the *m*/*z* range of 400 to 1000. PHMG oligomer peaks are labeled following the notation nomenclature shown in Figure 1. Inset: an enlarged mass spectrum showing a 4 Da spacing between the unlabeled C_3_ and labeled internal standard C_3_ * oligomer peaks.

Figure 3 shows a representative MALDI-TOF mass spectrum for one of the Oxy Saksak HD brand samples spiked with the 1000 ppm ^13^C PHMG internal standard. As clearly denoted with A*_l_*, B*_m_*, and C*_n_* series oligomer isomers, the PHMG oligomers were identified (Figure 1). These isomers, which were made naturally through a synthetic procedure, have different oligomer end groups. Each series of the PHMG oligomers was found to have a 141 Da spacing between the neighboring series oligomers, for example, A_3_ (*m*/*z* 441.4) and A_4_ (*m*/*z* 582.5), consistent with the reported PHMG unit monomer mass value [35]. It is also notable that in the insert of Figure 2, the ^13^C labeled internal standard peak (e.g., C_3_ *), appears with the *m*/*z* gap of 4 Da from the counterpart PHMG oligomer isomer peak C_3_; in each monomer unit, four carbons were labeled with ^13^C. By comparing the relative abundance ratios between the peaks unlabeled and labeled (e.g., C_3_ and C_3_ *), the amount of the PHMG oligomers in the sample can be roughly estimated, which in this case is 1800 ppm.

### 4.4. Calibration Curve

**Figure 4:**
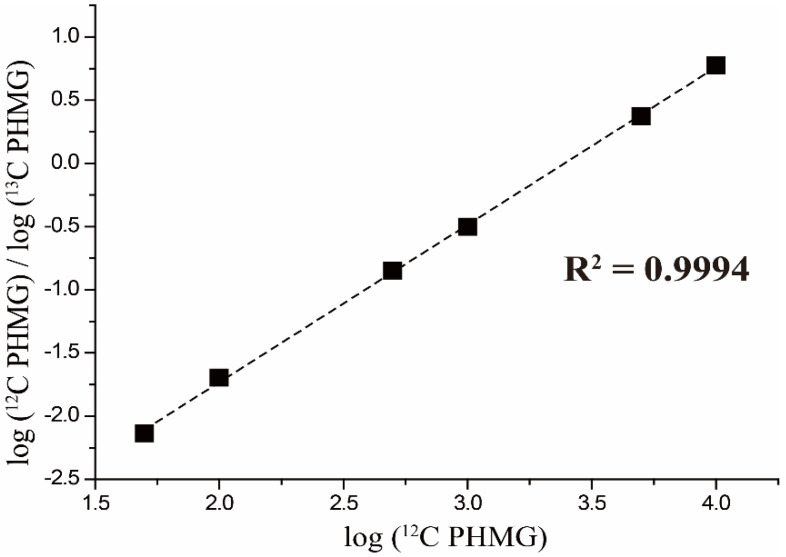
A calibration curve for PHMG in the range of 50–10,000 ppm.

A calibration curve was constructed for PHMG in the range of 50–10,000 ppm using six concentration points. The linearity of the calibration plot was satisfactory with a squared correlation coefficient (R^2^ = 0.9994) (Figure 4). The precision determined by calculating the coefficient of variation (CV, %) of replicates within runs on one day (intra-day) was well below 15% for a wide range of PHMG concentrations. The accuracy (relative error) obtained by calculating the percent deviation from the nominal concentration was mostly within 10% of the nominal values. The recovery efficiency was determined to be 73%; some analytes were lost during the sample purification procedure.

### 4.5. Average Molecular Mass of PHMG

The number-average (*M_n_*) and weight-average (*M_w_*) molecular masses (or weights) were determined using the following Equations (1) and (2) [36].

(1) $$M_{n}=\underset{i}{\sum }N_{i}M_{i}\;/\;\underset{i}{\sum }N_{i}$$

(2) $$M_{w}=\underset{i}{\sum }N_{i}{M_{i}}^{2}\;/\;\underset{i}{\sum }N_{i}M_{i}$$

where *N_i_* and *M_i_* refer to the number (here, abundance) of individual molecule (or peaks) and the individual molecular weight, respectively. The number-average molecular masses (weights) observed for the samples under examination were in the range of 422.0–546.9, corresponding to the 3–4 mers of PHMG. Because larger oligomer (polymer) molecules in a sample weigh more than smaller molecules, *M_w_* should always have a higher value than *M_n_* (i.e., *M_w_* > *M_n_*).

### 4.6. Degree of Polymerization of PHMG

The degree of polymerization is the number of monomeric units in a polymer or oligomer. For the given polymer or oligomer, the number- or weight-average degree of polymerization can be calculated using the following Equation (3).

(3) $$Degree\;of\;polymerization=M_{n}\;or\;M_{w}\;/\;M_{o}$$

where *M_o_* is the molecular weight of the monomer unit, which in this case is 141 g/mol.

### 4.7. Data Analysis

The physicochemical properties of PHMG, such as the concentration of PHMG dissolved in HD brands, molecular weight, and polymerization level, were shown along with the HD brand and year of manufacture. The samples with concentrations below the limit of quantitation (LOQ) were excluded. Descriptive analyses were performed using STATA 12.0 (STATA Corp, College Station, TX, USA) and R software (ver. 3.6.1, The R Foundation for Statistical Computing, Vienna, Austria).

## 5. Conclusions

This study found that the degree of PHMG oligomerization was in the range of two to four based on the average molecular weight and the degree of polymerization, and that the PHMGs used in the humidifier could be regarded as oligomers. Even though our results for the physicochemical properties of PHMG may not be representative of all HD brands containing PHMG, since HD brands vary by year of manufacture and type of HD brand, this study assumed that the highest proportion of HDLI caused by the PHMG in Korea may be related to several physicochemical properties, including high concentrations, low molecular weights with an oligomeric nature, and a low polymerization degree of PHMG. Further study is needed to examine how several physicochemical characteristics of PHMGs, including the molecular mass and level of HMDA, can be associated with HDLI.

## Figures and Tables

**Table 1:** Major brands of humidifier disinfectants containing PHMG.

Brand Name	Sales Period	Total Sales Volume (mL/each) [13]	Cases of HDLI ^1^
Oxy Saksak (Old and New versions)	2000–2011	9,561,151 of 500 mL	Yes
Lotte Wiselect	2006–2011	110,283 of 1000 mL	Yes
Homeplus	2004–2011	296,950 of 550 mL	Yes
Atorganic	2009–2011	No information	Yes
Cefu	2009–2011	No information	Yes
Vegetable Home Clean Up	2009–2011	106,943 of 1000 mL	No

^1^ The number of humidifier disinfectants associated with lung injury (HDLI) by the type of HD brand were reported elsewhere [4]. No HDLI cases were found among people who used other HD products not listed in this table.

**Table 2:** PHMG concentrations by the year of manufacture and type of humidifier brand.

Brand Name	Year	Number of Sample(s)	Concentration (ppm)		
			Mean	SD	Range
**Cleanland**	**NI**	1	1500.0	N/A	N/A
Oxy Saksak New	2004	3	3500.0	1479.9	2500–5200
	2005	7	2400.0	1724.3	900–5900
	2006	8	1431.3	820.1	670–3100
	2007	7	2382.9	1051.4	280–3500
	2008	4	1725.0	206.2	1500–2000
	2009	7	1914.3	803.0	1100–2800
	2010	14	2707.1	2025.0	1100–9000
	2011	14	1595.7	592.2	740–2600
	NI	15	1737.3	964.2	320–3900
	Subtotal	79	2051.0	1293.0	280–9000
Oxy	2001	1	2700.0	N/A	N/A
	2002	1	3700.0	N/A	N/A
	2003	1	3000.0	N/A	N/A
	NI	1	5000.0	N/A	N/A
	Subtotal	4	3600.0	1023.1	2700–5000
Vegetable Home Clean Up	2009	1	160.0	N/A	N/A
	2010	3	12,600.0	7607.9	4800–20,000
	NI	3	20,666.7	5859.5	14,000–25,000
	Subtotal	7	14,280.0	9261.4	160–25,000
Cefu	2011	2	19,700.0	24,748.7	2200–37,200
	NI	1	1600.0	-	1600
	Subtotal	3	13,666.7	20,382.7	1600–37,200
Atorganic	NI	1	560.0	N/A	N/A
Lotte Wiselect	2006	2	660.0	84.9	600–7200
	2008	1	690.0	N/A	N/A
	2010	3	1146.7	185.8	940–1300
	2011	1	1500.0	N/A	N/A
	Subtotal	7	992.9	345.9	600–1500
Homeplus	2009	1	1200.0	N/A	N/A
	2011	6	1800.0	1143.7	1000–4000
	NI	1	2700.0	N/A	N/A
	Subtotal	8	1837.5	1048.7	1000–4000
Total		110	3100.9	5102.8	160–37,200

Abbreviations: SD, (arithmetic) standard deviation; N/A, not applicable; NI, no information.

**Table 3:** The numbers (*M_n_*) and weights (*M_w_*) of average molecular masses of PHMG by brand and year manufactured.

Brand Name	Year	Number of Sample (s)	Average Molecular Weight (g/mol)					
			Number (Mn)			Weight (Mw)		
			Mean	SD	Range	Mean	SD	Range
Cleanland	NI	1	422.0	-	422.0	441.0	-	441.0
Oxy Saksak New	2004	3	535.0	22.4	519.0–560.6	551.0	23.5	535–578
	2005	7	524.8	21.7	481.0–541.1	544.1	14.9	521–558
	2006	8	503.2	53.4	435.0–560.8	523.4	48.7	460–578
	2007	7	534.4	14.3	518.5–560.3	550.0	15.9	531–578
	2008	4	534.2	10.9	519.5–545.7	550.5	11.1	536–563
	2009	7	501.9	37.4	437.0–539.0	525.0	29.6	469–554
	2010	14	521.3	41.7	448.0–613.0	545.1	45.9	480–678
	2011	14	510.3	46.1	448.0–562.2	532.4	39.2	480–581
	NI	15	533.1	7.3	520.3–545.9	549.5	7.8	537–564
	Subtotal	79	520.7	35.1	435.0–613.0	540.6	32.5	460–678
Oxy	2001	1	465.0	N/A	N/A	501.0	N/A	N/A
	2002	1	527.4	N/A	N/A	543.0	N/A	N/A
	2003	1	532.7	N/A	N/A	547.0	N/A	N/A
	NI	1	531.7	N/A	N/A	546.0	N/A	N/A
	Subtotal	4	514.2	32.9	465.0–532.7	534.3	22.2	501–547
Vegetable Home Clean Up	2009	1	483.0	N/A	N/A	483.0	N/A	N/A
	2010	3	542.4	38.9	515.2–587.0	562.7	43.7	529–612
	NI	3	516.7	1.4	515.4–518.3	530.0	1.0	529–531
	Subtotal	7	522.9	31.3	483.0–587.0	537.3	38.4	483–612
Cefu	2011	2	516.0	2.5	514.2–517.7	528.5	0.7	528–529
	NI	1	472.0	N/A	N/A	508.0	N/A	N/A
	Subtotal	3	501.3	25.4	472.0–517.7	521.7	11.8	508–529
Atorganic	NI	1	546.9	N/A	N/A	562.0	N/A	N/A
Lotte Wiselect	2006	2	531.4	7.1	526.4–536.5	548.0	8.5	542–554
	2008	1	484.0	N/A	N/A	522.0	N/A	N/A
	2010	3	517.5	39.1	475.0–551.9	540.3	29.0	511–569
	2011	1	533.0	N/A	N/A	549.0	N/A	N/A
	Subtotal	7	518.9	28.4	475.0–551.9	541.1	19.5	511–569
Homeplus	2009	1	444.0	N/A	N/A	475.0	N/A	N/A
	2011	6	493.9	41.1	446.0–532.7	522.0	28.4	484–549
	NI	1	527.0	N/A	N/A	542.0	N/A	N/A
	Subtotal	8	491.8	41.4	444.0–532.7	518.6	30.6	475–549
Total		110	517.2	35.9	422.0–613.0	537.3	32.7	441–678

Abbreviations: SD, (arithmetic) standard deviation; NI, no information; N/A, not applicable.

**Table 4:** The levels of polymerization of PHMG by HD brand.

Brand Name	Year	Number of Sample(s)	Mean	SD	Range
Cleanland	NI	1	2.05	-	2.05
Oxy Saksak New	2004	3	3.79	0.16	3.68–3.98
	2005	7	3.57	0.55	2.33–3.84
	2006	8	3.20	0.88	2.11–3.98
	2007	7	3.79	0.10	3.68–3.97
	2008	4	3.79	0.08	3.68–3.87
	2009	7	3.11	0.80	2.12–3.82
	2010	14	3.38	0.66	2.17–3.93
	2011	14	3.26	0.83	2.16–3.99
	NI	15	3.78	0.05	3.69–3.87
	Subtotal	79	3.48	0.63	2.11–3.99
Oxy	2001	1	2.26	N/A	N/A
	2002	1	3.74	N/A	N/A
	2003	1	3.78	N/A	N/A
	NI	1	3.77	N/A	N/A
	Subtotal	4	3.39	0.75	2.26–3.78
Vegetable Home Clean up	2009	1	3.43	N/A	N/A
	2010	3	3.02	0.57	2.55–3.65
	NI	3	3.66	0.01	3.66–3.68
	Subtotal	7	3.35	0.46	2.55–3.68
Cefu	2011	2	3.66	0.02	3.65–3.67
	NI	1	2.29	N/A	N/A
	Subtotal	3	3.20	0.79	2.29–3.67
Atorganic	NI	1	3.88	N/A	N/A
Lotte Wiselect	2006	2	3.77	0.05	3.73–3.80
	2008	1	2.35	NA	NA
	2010	3	3.31	0.88	2.30–3.91
	2011	1	3.78	NA	NA
	Subtotal	7	3.37	0.72	2.30–3.91
Homeplus	2009	1	2.15	NA	NA
	2011	6	2.99	0.85	2.16–3.78
	NI	1	3.74	NA	NA
	Subtotal	8	2.98	0.83	2.15–3.78
Total		110	3.41	0.66	2.05–3.99

Abbreviations: SD, (arithmetic) standard deviation; NI, no information; N/A, not applicable.
